# Deep sequencing of the prothoracic gland transcriptome reveals new players in insect ecdysteroidogenesis

**DOI:** 10.1371/journal.pone.0172951

**Published:** 2017-03-03

**Authors:** Takayoshi Nakaoka, Masatoshi Iga, Tetsuya Yamada, Ikumi Koujima, Mika Takeshima, Xiangying Zhou, Yutaka Suzuki, Mari H. Ogihara, Hiroshi Kataoka

**Affiliations:** 1 Department of Integrated Biosciences, Graduate School of Frontier Sciences, The University of Tokyo, Kashiwa, Chiba Japan; 2 Department of Computational Biology and Medical Sciences, Graduate School of Frontier Sciences, The University of Tokyo, Kashiwa, Chiba Japan; Institute of Plant Physiology and Ecology Shanghai Institutes for Biological Sciences, CHINA

## Abstract

Ecdysteroids are steroid hormones that induce molting and determine developmental timing in arthropods. In insect larva, the prothoracic gland (PG) is a major organ for ecdysone synthesis and release. Released ecdysone is converted into the active form, 20-hydroxyecdysone (20E) in the peripheral tissues. All processes from ecdysone synthesis and release from the PG to its conversion to 20E are called ecdysteroidogenesis and are under the regulation of numerous factors expressed in the PG and peripheral tissues. Classical genetic approaches and recent transcriptomic screening in the PG identified several genes responsible for ecdysone synthesis and release, whereas the regulatory mechanism remains largely unknown. We analyzed RNA-seq data of the silkworm *Bombyx mori* PG and employed the fruit fly *Drosophila melanogaster* GAL4/UAS binary RNAi system to comprehensively screen for genes involved in ecdysone synthesis and/or release. We found that the genes encoding δ-aminolevulinic acid synthase (*CG3017*/*alas*) and putative NAD kinase (*CG33156*) were highly expressed in the PG of both *B*. *mori* and *D*. *melanogaster*. Neither *alas* nor *CG33156* RNAi-induced larvae could enter into the pupal stage, and they had a lower abundance of the active form ecdysteroids in their prolonged larval stage. These results demonstrated that *alas* and *CG33156* are indispensable for ecdysteroidogenesis.

## Introduction

Steroid hormones play crucial roles in the regulation of many biological processes in vertebrates and invertebrates. In insects, growth is controlled by molting and metamorphosis, and characteristic developmental events are regulated by steroid hormones called ecdysteroids. During the larval stage, ecdysone is synthesized from cholesterol through serial oxidation/hydroxylation steps in the prothoracic gland (PG) and secreted into the hemolymph [1–4]. The released ecdysone is converted to the biologically active form of ecdysone, 20-hydroxyecdysone (20E), in peripheral tissues [5]. The sequence of biological processes from ecdysone synthesis in the PG to 20E production in the peripheral tissues is called ecdysteroidogenesis. As the timing of molting and metamorphosis are determined by ecdysone production and secretion from the PG, it is important to understand the regulatory mechanisms of ecdysteroidogenesis.

Genes expressed predominantly in the PG are expected to be involved in ecdysone synthesis and release. In the last decade, analyses of the PG gene expression using a plethora of molecular techniques has successfully identified genes that are indispensable for ecdysone biosynthesis. For example, Rieske-domain oxygenase Neverland, which is an enzyme for 7, 8-dehydrogenation of cholesterol, Cyp306a1/phantom, and Cyp307a1/spook, which are collectively called Halloween genes and encode cytochrome P450 monooxygenases, were identified via cDNA microarray analyses or fluorescent differential display [6–8]. In addition to ecdysteroidogenic enzymes, recent studies showed additional factors made PG as an ecdysteroidogenic organ, such as an ecdysone-specific ABC transporter, *atet*, and a transcription factor, *ooija board* [9, 10].

High-throughput RNA sequencing (RNA-seq) is an effective and quantitative method for transcriptomic analysis [11]. On an RNA-seq platform, a huge number of sequences in a cDNA library from a tissue of interest are determined. RNA-seq has been applied to characterize distinct tissues in various insect species [12]. To identify novel factor(s) responsible for ecdysone synthesis and/or release, we have conducted RNA-seq analysis of the PG of the silkworm *Bombyx mori* [13]. Focused screening of G protein-coupled receptors (GPCRs) in the PG transcriptome revealed a GPCR, BNGR-B2, as a receptor for the pigment-dispersing factor that enhances ecdysone synthesis and release in the PG. However, further players are considered essential for PG ecdysteroidogenesis and they would be discovered in the RNA-seq data.

Here we report detailed analysis of the RNA-seq data and the use of an additional screening method, the GAL4/UAS-based RNA interference (RNAi), in *Drosophila* to reveal genes responsible for ecdysteroidogenesis in the PG. We analyzed the expression profile of 21,302 genes predicted in the *Bombyx mori* genome and transcriptomically screened for identified genes expressed more in the PG than in the brain. Screening via *Drosophila* RNAi highlighted two genes, *alas* and *CG33156*, as candidate genes responsible for ecdysteroidogenesis in the PG. Knockdown of *alas* or *CG33156* in the PG caused developmental arrest at the third instar larva with no morphogenetic abnormality, along with a low abundance of 20E during an extended larval phase. These results suggested that *alas* and *CG33156* are required for proper ecdysone synthesis and/or release in insect larval development.

## Materials and methods

### Animals

*Drosophila melanogaster* strains were maintained on standard cornmeal medium at 25°C under 12-h light:12-h dark photoperiod. *yw* was used as the wild type fly strain. *UAS*-*Dcr2*; *phm*-*GAL4*, and *UAS*-*Dcr2*; *2*-*286*-*GAL4* were used for in vivo RNAi. Virgin females with *UAS*-*Dcr2*; *phm*-*GAL4* or *UAS*-*Dcr2*; *2*-*286*-*GAL4* were crossed to UAS-Inverted Repeat (IR) males. Females were allowed to lay eggs overnight, and the numbers of adult or pupae were counted until day 14 or day 8, respectively. Flies with UAS-IR constructs were obtained from the Vienna Drosophila RNAi Center (VDRC) and the National Institute of Genetics (NIG-FLY). The *UAS*-*mCD8*::*GFP* line was obtained from the Bloomington Drosophila Stock Center.

### Transcriptomic analysis of silkworm PG

RNA-seq of *Bombyx mori* PG and brain in wandering larvae were previously performed as outlined in [13] (Accession number: DRA002282). Sequence reads were mapped to the reference model transcripts according to the *B*. *mori* genome annotations (http://silkworm.big.ac.cn/jsp/download.jsp). The number of reads uniquely aligned to the genome without any mismatches were digitally counted. Parts per million (ppm) to the total mapped reads were calculated for each gene, and the ppm ratio of PG to the brain was calculated to compare gene expression levels.

### Quantitative RT-PCR

The ring gland (RG), central nervous system, salivary gland, fat body, Malpighian tubules, trachea, midgut, and epidermis were dissected from wandering *yw* third instar larvae. For each sample, tissues were collected from 40 larvae, and three independent, biological replicates were performed. Total RNA was isolated using the TRIzol reagent, and the isolated RNA was treated with RQ DNaseI (Promega) to digest genomic DNA. Reverse-transcription was performed using the PrimeScript RT reagent kit (Perfect Real Time) (TaKaRa) with both random hexamers and oligo (dT) primer, following manufacturers’ instructions. Real-time PCR was performed with the Thermal Cycler Dice and SYBR Premix ExTaq II (TaKaRa). The expression levels were normalized to *rp49*, and the ΔΔCt method was used to calculate the expression ratio for each gene. Averages of triplicate samples were calculated for each tissue, and the highest expression level among tissues was set to 1. Samples with Ct values over 38 were excluded. The primers used in this study are listed in S1 Table.

### *In situ* hybridization

Nucleotide fragments corresponding to candidate genes were amplified by RT-PCR and subcloned into the pGEM-T vector (Promega). Primers used are listed in S1 Table. Sense or antisense RNA probes were synthesized using the DIG labeling mix (Roche) with T7 and SP6 RNA polymerase. RG-brain complexes were dissected from *yw* wandering larvae and fixed in 4% cold formaldehyde/PBS. Subsequent procedures were carried out accordingly as previously reported [14] but with some modifications. To detect RNA probes, the specimens were incubated in PBS with 0.3% Triton X-100 containing anti-DIG antibody conjugated with alkaline phosphatase, and 4-nitroblue tetrazolium chloride-5-bromo-4-chloro-3-indolyl-phosphate (NBT-BCIP) was used as a chromogenic substrate.

### Measurement of ecdysteroids

Females with *phm-GAL4/ TM3 Ser Kr-GFP* were crossed to males homozygous for *UAS-IR* and allowed to lay eggs on standard cornmeal food for 6 h. GFP larvae (negative control), with genotype *UAS-IR and TM3 Ser Kr-GFP*, were collected at 96 and 120 (±3) h after egg laying (h AEL). Both wandering larvae and white pupae were observed at AEL 120 in the controls, and therefore, both were collected at 120 h AEL. On the other hand, GFP negative larvae, with genotype *phm-GAL4* and *UAS-IR*, were collected at 96, 120, and 144 (±3) h AEL. Five pupae or ten larvae were collected for each sample in a 1.5-ml tube and stored at -20°C until further use. Collected larvae were homogenized with a close-fitting plastic pestle and sonicated for 10 min in 200 μl of buffer containing 50 mM Tris-HCl, pH 7.5, 150 mM NaCl, and 2 mM EGTA. Steroids were extracted from the samples with 1-butanol (Wako). Two-fold volumes of 1-butanol were added to sonicated samples and shaken vigorously for 5 min. After centrifuging at 1,000 ×*g*, supernatants were transferred to a new tube. Steroid extraction using 1-butanol was repeated two more times, and the solution was pooled in the same tube. The solvent was evaporated in a centrifugal evaporator, and the residue was dissolved in 50 μl of methanol (Wako). Dissolved samples were shaken vigorously for 5 min and sonicated for 10 min. After centrifuging at 17,800 ×*g*, the supernatant was transferred to a glass vial. Ten microliters were injected into a LC-MS/MS system to measure steroid amount. Calibration curves were generated with a 0.5–500 ng/ml dilution series of ecdysone, 20E and Makisterone A. Conditions for the LC-MS/MS analysis, Prominence gradient HPLC system (Shimadzu), and the triple quadrupole QTRAP 5500 mass spectrometer (AB SCIEX) are described in [15]. Conditions to detect Makisterone A are shown in S2 Table. Makisterone A was purchased from Santa Cruz Biotechnologies. Ecdysone and 20-hydroxyecdysone were purchased from Sigma-Aldrich.

### Morphology of RG

The offspring of *UAS*-*Dcr2*; *UAS-mCD8*::*GFP*; *phm-GAL4/TM6B Tb*, and *UAS-IR/UAS-IR* flies were reared on standard media, and RG-brain complexes were dissected from day 5 and 8. RG-brain complexes were then observed using a U-MNIBA2 fluorescent microscope (BX-60, Olympus).

## Results

### Screening of genes expressed preferentially in the *B*. *mori* PG

The PG is an organ that specifically synthesizes and secretes ecdysone; therefore, higher expression of responsible genes for ecdysteroidogenesis in the PG was expected as compared to other tissues. To identify such genes, we performed RNA-seq using the PG and brain of *B*. *mori* wandering larva [13]. The brain was used as a non-steroidogenic control organ. Fifty million single-end reads of 36 base pairs were determined per organ, and 20,084,675 reads from the PG (total of 42,418,141 reads) and 18,628,667 reads from the brain (total of 47,649,669 reads) were uniquely aligned to the *B*. *mori* genome (http://silkworm.big.ac.cn/jsp/download.jsp). The number of reads aligned to a gene was counted, and ppm of the mapped reads to all of the uniquely aligned reads was calculated to estimate the expression level of each gene. The ratios of ppm in the PG to those in the brain were calculated to compare gene expression levels. To evaluate the quantification performance of our RNA-seq, we analyzed the expression ratios of some housekeeping genes as well. In the top 30 genes expressed in the PG, we found 15 genes encoding ribosomal proteins (S3 Table). The mean and standard deviation of expression ratios of these 15 ribosomal protein genes were 1.22 and 0.144, respectively. In addition, the expression ratios of three elongation factor genes, *EF1α*, *EF1γ*, and *EF2* were 1.87, 1.22, and 1.63, respectively (S3 Table). These results suggested that comparing ppm of any gene of interest was suitable to evaluate differential gene expression in the PG and brain.

**Fig 1:**
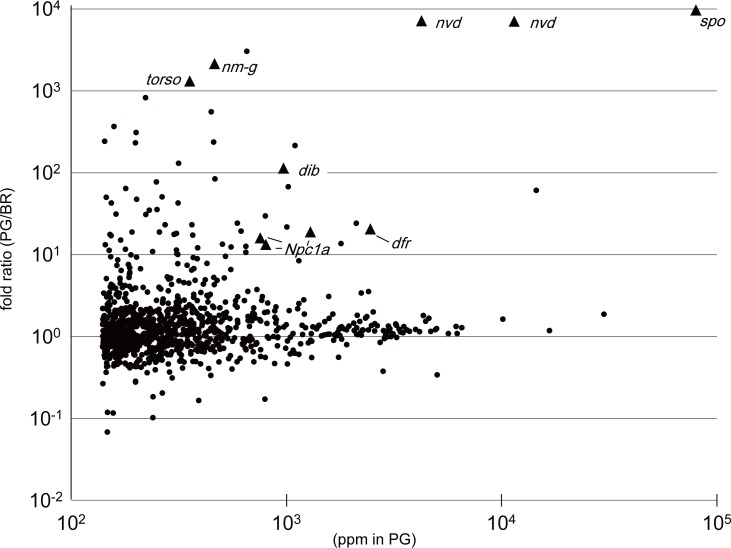
Expression profiles of the top 1,000 genes expressed in the PG. Each data point represents ppm in the PG (x-axis) and relative expression ratio compared with the brain (y-axis) of each gene. Genes that are known to be involved in ecdysone biosynthesis are represented by triangles.

Next, we searched for genes potentially involved in ecdysteroidogenesis in the PG. Of the 21,302 genes predicted in the genome database, the top 1,000 highly expressed genes covered a significant portion of the PG transcriptome (13,957,412 reads; 69.5% of all mapped sequence reads). We searched gene fold-ratios >10 in the top 1,000 genes. Following this criterion, we obtained 62 candidate genes that showed preferential expression in the PG (Fig 1, Table 1). Previously reported ecdysteroidogenic genes, such as *neverland* (Bmb000191, Bmb028931) [8], *spook* (Bmb008079) [6, 16], *short*-*chain dehydrogenase*/*reductase* (known as *nm-g*/*shroud* Bmb002129) [17], *disembodied* (Bmb019913) [18, 19], *torso* (Bmb009410) [20], *silk gland factor3*/*ventral vein lacking* (Bmb024277) [21], and *Npc1a* (Bmb016744, Bmb016745, Bmb020860) [22] were in this candidate list (Table 1, Fig 1). Due to an incomplete silkworm genome database, RNA-seq reads mapped to different contigs derived from the same genes. For example, sequence reads for *neverland* mapped to Bmb028931 and Bmb000191 that encoded amino acid residues 114–301 and 302–453 of the Neverland protein (NP_001037626.1). Although the mapping was not integral, both split sequences were detected as genes specifically expressed in the PG. Therefore, other responsible genes for ecdysone synthesis and release possibly existed in the list of candidate genes.

**Table 1:** List of genes expressed >10 times in the PG than in the brain.

Gene Name	ppm	RatioPG/Br	Description	Accession #	Fly homolog
Bmb012212	552.0	12.4	actin [Aedes aegypti]	XP_001659963.1	CG10067/act57B
Bmb025648	200.9	47.4	aminopeptidase N-12 [Bombyx mori]	AFK85028.1	CG11956/SP1029
Bmb007487	2108.3	24.1	chitinase-related protein 1 [Danaus plexippus]	EHJ65741.1	CG18140/cht3
Bmb040752	248.2	77.1	chitinase-related protein 1 precursor [Bombyx mori]	NP_001036861.1	CG18140/cht3
Bmb024372	263.7	50.6	cytochrome P450 18a1 [Bombyx mori]	NP_001077078.1	CG6816/Cyp18a1
Bmb019913	966.7	114.0	cytochrome P450 302A1 [Bombyx mori]	NP_001036953.1	CG12028/dib
Bmb008079	79847.5	9721.9	cytochrome P450, family 307, subfamily a, polypeptide 1 precursor [Bombyx mori]	NP_001104833.1	CG10594/spo
Bmb007257	314.9	130.4	endonuclease-reverse transcriptase [Bombyx mori]	ADI61816.1	--
Bmb002562	385.8	12.1	inositol 1 [Papilio xuthus]	BAM18672.1	CG4026/IP3K1
Bmb012355	14470.0	60.8	insulin-related peptide binding protein precursor [Bombyx mori]	NP_001040473.1	CG15009/ImpL2
Bmb035169	796.4	29.6	insulin-related peptide binding protein precursor [Bombyx mori]	NP_001239349.1	CG15009/ImpL2
Bmb022329	307.5	18.1	juvenile hormone esterase [Bombyx mori]	AAR37335.1	CG10175
Bmb021298	308.8	10.3	malate dehydrogenase [Bombyx mori]	NP_001093280.1	CG5889/Men-b
Bmb031565	221.1	823.6	neuropeptide receptor A34 [Bombyx mori]	NP_001127750.1	CG30340
Bmb000191	11416.7	7089.2	neverland [Bombyx mori]	NP_001037626.1	CG40050/nvd
Bmb028931	4236.1	7173.9	neverland [Bombyx mori]	NP_001037626.1	CG40050/nvd
Bmb024993	646.6	12.6	no hit	--	--
Bmb038048	178.8	64.1	no hit	--	--
Bmb021395	1788.7	13.6	PREDICTED: 5-aminolevulinate synthase, nonspecific, mitochondrial-like [Bombyx mori]	XP_004922915.1	CG3017/Alas
Bmb005099	457.7	236.8	PREDICTED: adenylate cyclase type 5-like [Bombyx mori]	XP_004932850.1	CG42513
Bmb005846	1095.1	214.7	PREDICTED: adenylate cyclase type 5-like [Bombyx mori]	XP_004932850.1	CG42513
Bmb037415	157.6	366.9	PREDICTED: adenylate cyclase type 5-like [Bombyx mori]	XP_004932850.1	CG42513
Bmb030773	142.7	241.7	PREDICTED: bile salt-activated lipase-like isoform X1 [Bombyx mori]	XP_004924253.1	CG10175
Bmb004665	272.4	23.2	PREDICTED: chaoptin-like [Bombyx mori]	XP_004923576.1	CG1744/chp
Bmb034135	191.1	18.8	PREDICTED: chaoptin-like [Bombyx mori]	XP_004923576.1	CG1744/chp
Bmb040810	503.4	13.4	PREDICTED: chaoptin-like [Bombyx mori]	XP_004923576.1	CG1744/chp
Bmb007256	198.3	230.9	PREDICTED: ecdysone-inducible protein E75-like [Bombyx mori]	XP_004924954.1	CG18023/Eip78
Bmb021498	256.3	18.8	PREDICTED: facilitated trehalose transporter Tret1-like [Bombyx mori]	XP_004923499.1	CG8234/Tret1-2
Bmb025408	230.0	34.8	PREDICTED: FH1/FH2 domain-containing protein 3-like [Bombyx mori]	XP_004929944.1	CG42610/Fhos
Bmb038593	169.3	15.0	PREDICTED: GRAM domain-containing protein 1B-like [Bombyx mori]	XP_004929648.1	--
Bmb001251	590.6	24.2	PREDICTED: GRAM domain-containing protein 1B-like [Bombyx mori]	XP_004930811.1	CG34394
Bmb005705	446.1	554.0	PREDICTED: lipase member H-like [Bombyx mori]	XP_004931604.1	--
Bmb002715	307.8	11.4	PREDICTED: LOW QUALITY PROTEIN: papilin-like [Bombyx mori]	XP_004921811.1	--
Bmb015275	313.1	42.6	PREDICTED: LOW QUALITY PROTEIN: papilin-like [Bombyx mori]	XP_004921811.1	CG33103/Ppn
Bmb021601	1003.6	21.7	PREDICTED: LOW QUALITY PROTEIN: papilin-like [Bombyx mori]	XP_004921811.1	CG33103/Ppn
Bmb000943	238.4	10.9	PREDICTED: LOW QUALITY PROTEIN: titin-like [Apis florea]	XP_003693102.1	CG1915/sls
Bmb036727	300.0	17.7	PREDICTED: luciferin 4-monooxygenase-like, partial [Bombyx mori]	XP_004927714.1	CG8834
Bmb000190	652.9	3040.8	PREDICTED: multiple coagulation factor deficiency protein 2 homolog isoform X2 [Bombyx mori]	XP_004922199.1	--
Bmb018637	312.0	10.0	PREDICTED: NAD kinase-like isoform X2 [Bombyx mori]	XP_004926312.1	CG33156
Bmb016744	1289.5	19.0	PREDICTED: niemann-Pick C1 protein-like isoform X1 [Bombyx mori]	XP_004924425.1	CG5722/Npc1a
Bmb016745	753.7	16.0	PREDICTED: niemann-Pick C1 protein-like isoform X1 [Bombyx mori]	XP_004924425.1	CG5722/Npc1a
Bmb020860	799.9	13.3	PREDICTED: niemann-Pick C1 protein-like isoform X1 [Bombyx mori]	XP_004924425.1	CG5722/Npc1a
Bmb000196	199.7	310.0	PREDICTED: nose resistant to fluoxetine protein 6-like [Bombyx mori]	XP_004933619.1	CG3106
Bmb030828	363.3	23.2	PREDICTED: phosphatidylinositol 3-kinase catalytic subunit type 3-like [Bombyx mori]	XP_004925089.1	CG5373/Pi3K59F
Bmb036448	646.9	10.6	PREDICTED: pogo transposable element with ZNF domain-like [Bombyx mori]	XP_004922578.1	--
Bmb020216	178.1	11.6	PREDICTED: protein FAM135A-like [Bombyx mori]	XP_004931668.1	CG32333
Bmb002130	151.1	17.6	PREDICTED: serine protease easter-like [Bombyx mori]	XP_004932592.1	CG16705/SPE
Bmb008527	145.1	50.1	PREDICTED: serine/threonine-protein kinase prpf4B-like [Bombyx mori]	XP_004921796.1	CG2054/cht2
Bmb033191	143.7	13.2	PREDICTED: shootin-1-like [Bombyx mori]	XP_004933010.1	--
Bmb025563	366.1	17.3	PREDICTED: solute carrier family 2, facilitated glucose transporter member 1-like [Bombyx mori]	XP_004923552.1	CG1086/Glut1
Bmb003383	464.6	84.0	PREDICTED: solute carrier organic anion transporter family member 3A1-like [Bombyx mori]	XP_004932455.1	CG7571/Oatp74D
Bmb030561	1017.9	67.2	PREDICTED: solute carrier organic anion transporter family member 3A1-like [Bombyx mori]	XP_004932455.1	CG7571/Oatp74D
Bmb010938	249.7	35.5	PREDICTED: transmembrane protein nessy-like [Bombyx mori]	XP_004933932.1	CG9655/nes
Bmb004658	149.7	11.2	PREDICTED: ubiquitin-conjugating enzyme E2 R2-like [Bombyx mori]	XP_004922372.1	CG7656
Bmb002531	161.2	31.3	PREDICTED: uncharacterized protein LOC101738974 [Bombyx mori]	XP_004926099.1	CG8420
Bmb039058	221.7	30.8	putative trypsin-like serine protease [Danaus plexippus]	EHJ67268.1	CG7996/snk
Bmb002258	613.9	19.4	ras-related GTP-binding protein Rab3 [Bombyx mori]	NP_001037620.1	CG7576/rab3
Bmb038774	153.3	42.6	serine protease inhibitor 11 precursor [Bombyx mori]	NP_001139704.1	CG10913/Spn6
Bmb032080	152.8	17.0	serine protease inhibitor 3 precursor [Bombyx mori]	NP_001040318.1	CG11331/Spn27
Bmb002129	462.1	2152.3	short-chain dehydrogenase/reductase [Bombyx mori]	NP_001171333.1	CG12068/sro
Bmb024277	2449.4	20.6	silk gland factor 3 [Bombyx mori]	NP_001037456.2	CG10037/dfr
Bmb009410	354.3	1320.2	tyrosine-protein kinase receptor torso [Bombyx mori]	NP_001164049.1	CG1389/torso

### Phenotypic screening using the *Drosophila* GAL4/UAS RNAi system

Many of the ecdysteroidogenic enzymes and regulatory mechanisms for PG function are conserved between *Bombyx* and *Drosophila* [6, 7, 17, 20, 23]. The GAL4/UAS-based RNAi system in *Drosophila* is a suitable approach for comprehensive screening. Therefore, we used RNAi screening in *Drosophila* to assess the contribution of candidate genes identified in our *B*. *mori* RNA-seq analysis to ecdysone synthesis and/or release. *Drosophila* genes homologous to the *Bombyx* PG-preferential genes were searched via BLASTX against Fly Base (http://flybase.org/) with a cut-off value of 1e-5. Out of 62 genes obtained from the *Bombyx* RNA-seq expression profile, 11 genes were known to be involved in ecdysone biosynthesis or ecdysone metabolism as described above, and nine genes (*Bmb007257*, *Bmb024993*, *Bmb038043*, *Bmb038593*, *Bmb005705*, *Bmb002715*, *Bmb000190*, *Bmb036448*, and *Bmb033191*) had no homologous genes in the *Drosophila* genome (Table 1). After excluding the redundant *B*. *mori* genes (*Bmb022329*/*Bmb030773*, *Bmb012355*/*Bmb035169*, *Bmb007487*/*Bmb040752*, *Bmb015275*/*Bmb021601*, *Bmb003383*/*Bmb030561*, *Bmb005099*/*Bmb005846*/*Bmb037415*, and *Bmb004665*/*Bmb034135*/*Bmb040810*), 33 homologous genes in *Drosophila* were determined (Table 1). We obtained 53 transgenic lines carrying IR sequences corresponding to 27 out of the 33 candidate genes (S4 Table). To knockdown each target gene in the PG specifically, male flies with the IR construct were crossed to *UAS*-*Dcr2*; *phm-GAL4*/*TM3 Ser* females, and the number of adults emerged was counted. Out of 53 lines corresponding to the 27 genes tested, 11 lines corresponding to eight genes showed a significant decrease of adult flies as compared to controls (chi-square test, *p* < 0.01) (Table 2). The other six genes were not examined because RNAi lines were not available. To determine their lethal phase, the males were crossed to *Dcr2*; *phm-GAL4/TM6B Tb* females to observe if the RNAi strain with the homozygous UAS-IR genotype was viable. The RNAi larvae were fed for 7 days after oviposition, and their developmental stages were classified according to spiracle and mouth hook morphology. In all nine lines examined, we observed either no or significantly less (chi-square test, *p* < 0.01) pupae in which the target gene was disrupted (Table 2). *CG10913*/*Serpin6* (*Spn6*) RNAi animals died at the second instar larval stage. Other RNAi larvae could grow up to the third instar, but they could not form pupae except for some pupariated individuals. To confirm that the developmental abnormality did not result from unexpected expression of *phm-GAL4* other than in the PG, the UAS-IR males were crossed to another PG-expressing GAL4 driver line, *Dcr2*; *2-286-GAL4*/*TM6B Tb* females, and the number of pupae was counted. For all nine lines that showed developmental arrest by *phm-GAL4*, this same phenotype was observed when *2-286-GAL4* was used (Table 2). These results suggested that these genes play crucial roles in *Drosophila* molting or metamorphosis.

**Table 2:** Results of RNAi driven by *phm-GAL4* or *2-286-GAL4*.

					Dcr2; phm-GAL4/TM3 Ser		Dcr2; phm-GAL4/TM6B Tb		Dcr2; 2-286-GAL4/TM6B Tb		
					number of adult		number of pupa		number of pupa		
Gene Name	Ratio PG/BR	Description	Fly homolog	RNAi line	control	RNAi	control	RNAi	control	RNAi	lethal stage
Bmb021395	13.6	5-aminolevulinate synthase [Bombyx mori]	CG3017/alas	N 3017R1	148	0	129	0	23	0	L3
				N 3017R2	91	12	89	34	269	0	L3
Bmb018637	10.0	Predicted: NAD kinase-like isoform X2 [Bombyx mori]	CG33156	N 6152R3	60	0	104	0	48	0	L3
				N 6152R4	237	10	249	53	81	0	L3
Bmb000943	10.9	Predicted: titin-like [Apis florea]	CG1915/sls	V 47298	136	0	40	0	144	0	L2/L3
				V 47301	111	0	167	0	23	0	L2/L3
Bmb001251	24.2	Predicted: GRAM domain-containing protein 1B-like [Bombyx mori]	CG34394	V 23105	121	1	131	23	100	32	L3
Bmb002258	19.4	ras-related GTP-binding protein Rab3 [Bombyx mori]	CG7576/rab3	V 100787	91	0	156	0	98	1	L3
Bmb038774	42.6	serine protease inhibitor 11 precursor [Bombyx mori]	CG10913/Spn6	N 10913R1	203	1	152	0	42	0	L2
Bmb000196	310.0	Predicted: nose resistant to fluoxetine protein 6-like [Bombyx mori]	CG3106	V 42701	226	21	--	--	--	--	--
Bmb002531	31.3	Predicted: uncharacterized protein LOC101738974 [Bombyx mori]	CG8420	N 8420R2	305	45	--	--	--	--	--

In the “RNAi line” column, V and N indicate that the RNAi lines were obtained from VDRC and NIG-FLY, respectively.

### Expression analysis for candidate genes

**Fig 2:**
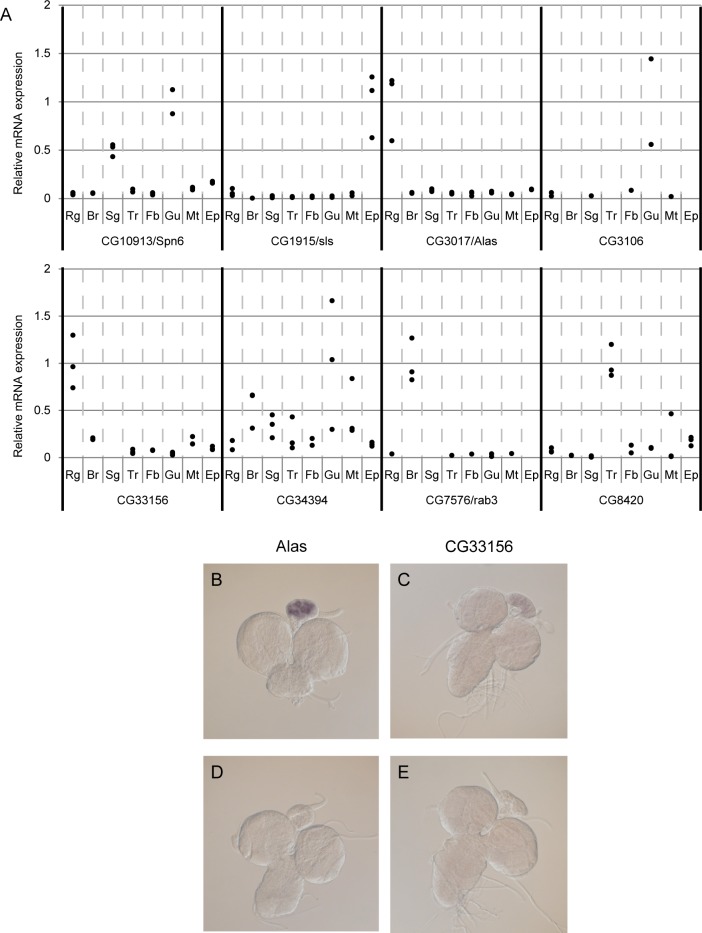
Expression analysis of candidate genes. (A) Quantitative real-time PCR for candidate genes in several tissues from wandering third instar larvae of *D*. *melanogaster*. Rg, ring gland; Br, brain with ventral nerve cord; Sg, salivary gland; Tr, trachea; Fb, fat body; Gu, Gut; Mt, Malpighian tubule; Ep, epidermis. The expression level of each gene was normalized using *rp49* as an endogenous control. Relative expression levels were determined using the ΔΔCt method. Among tissues examined, the highest average expression level was set to 1. (B-E) *In situ* hybridization analysis for candidate genes in the ring gland. a*las* and *CG33156* transcripts were detected with antisense probes but not with sense probes. B: *alas* antisense, C: *CG33156* antisense, D: *alas* sense, and E: *CG33156* sense.

Genes that specifically function in ecdysone synthesis or release are expected to be expressed in the RG, which is a complex organ mostly comprised of PG cells. To examine tissue distribution of candidate genes, the expression levels in several tissues (RG, central nervous system, salivary gland, trachea, fat body, gut, Malpighian tubule, and epidermis) of wandering *yw* third instar larvae were analyzed by real-time RT-PCR. *alas* and *CG33156* were expressed most predominantly in the RG (Fig 2A). However, other candidate genes did not show this RG-preferential expression pattern, even though knockdown of these genes caused developmental defects. In addition, whole mount *in situ* hybridization was conducted to confirm gene expression in the RG of wandering *yw* third instar larvae. Regarding *alas* and *CG33156*, we showed that antisense probe specific signals were detected in the RG (Fig 2B–2E), whereas no clear signals were detected with probes for the other six candidate genes (data not shown). *alas* and *CG33156* showed typical expression profiles for genes responsible for ecdysone synthesis or release, and while the other six genes might be expressed in the PG, they may be less expressed or temporally restricted during development.

### Ecdysteroid titers and RG morphology in RNAi larvae

**Fig 3:**
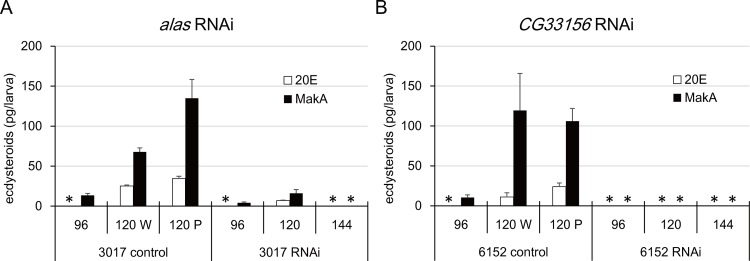
Ecdysteroids in *D*. *melanogaster* RNAi larvae. Whole body 20E and MakA titers of RNAi and control larvae were measured by LC-MS/MS. (A) 20E and MakA titers of *alas* RNAi animals. (B) 20E and MakA titers of *CG33156* RNAi animals. Data are from three or four biological replicates, which consisted of 10 larvae or 5 pupae for each data point. W: wandering larvae and P: prepupae. Values are mean ± S.E.M. and asterisks indicate under quantitation limit (2.5 pg/larva or 5 pg/pupa).

Since *alas* and *CG33156* were predominantly expressed in the RG and disrupting their expression in the PG resulted in the developmental arrest at the larval stage, loss of gene function was expected to cause defects in ecdysteroid synthesis and/or release. To test this hypothesis, we measured ecdysteroid titers in the larvae using an LC-MS/MS quantification method established previously [15]. In addition to ecdysone and 20E, the abundance of Makisterone A (MakA), which is another major ecdysteroid in *Drosophila* [24], was determined. In our experiments, all animals were third instar larvae at 96 h after egg laying (96 h AEL), while both wandering larvae and prepupae coexisted at 120 h AEL in the control group. Therefore, we examined ecdysone, 20E, and MakA titers at 96 h AEL feeding larvae, 120 h AEL in wandering larvae, and 120 h AEL in white prepupae from control groups. On the other hand, all larvae were still in the third instar at 96 h, 120 h, and 144 h AEL in the RNAi groups. Levels of 20E and MakA in the control groups were low during the feeding stage and increased with regular development. In contrast, these steroid titers in *alas* or *CG33156* RNAi larvae remained at basal levels up to 144 h AEL (Fig 3). On the other hand, ecdysone was not detected in any samples examined; therefore, it is not clear that Alas and CG33156 are responsible for ecdysone synthesis or release. These results suggested that PG function is critically disrupted by interfering with *alas* or *CG33156*, and particularly, their larval arrest phenotype resulted from 20E and MakA deficiency during the third instar larval stage.

**Fig 4:**
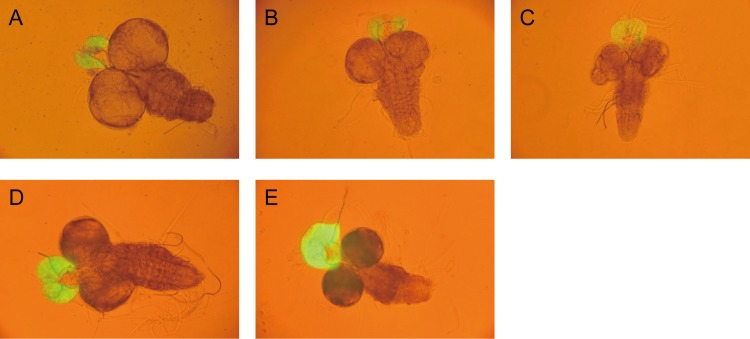
RG morphology of *D*. *melanogaster* RNAi larvae. RG from day 5 or day 8 larvae were visualized by mCD8::GFP under the *phm-GAL4* driver. (A) Representative image of the RG from day 5 control larva (*phm* > *mCD8*::*GFP*). (B) and (D) Representative images of the RG from day 5 and day 8 *alas* RNAi larvae (*phm* > *mCD8*::*GFP*, *alas* inverted repeat), respectively. (C) and (E) Representative images of the RG from day 5 and day 8 *CG33156* RNAi larvae, respectively (*phm* > *mCD8*::*GFP*, *CG33156* inverted repeat). The morphology of the RG of RNAi larvae did not differ from that of control larvae at day 5, but the RG of RNAi larvae became larger during their extended larval period.

To examine if the ecdysteroids depletion was due to the failure of RG organogenesis, the RGs of RNAi larvae were visualized by expressing *mCD8*::*GFP* under the control of *phm-GAL4*. RG morphology of *alas* or *CG33156* RNAi larvae was normal as compared to controls at day 5 in the third instar larval stage (Fig 4A–4C). These RNAi larvae continued feeding after control animals became pupae, and they grew larger in the extended larval stage up to 10 days. In the prolonged larval stage, the RGs of RNAi larvae hypertrophied at day 8 with growth of their bodies (Fig 4D and 4E). In addition, the GFP signal in the RG appeared to be stronger probably due to additional expression of GFP during the extended larval period. These results suggested that these genes affect neither differentiation nor growth of the RG cells.

## Discussion

In the present study, RNA-seq of target tissues unraveled many genes implicated in regulation of ecdysone synthesis and/or release as being expressed preferentially in the PG of *B*. *mori*. Most genes required for ecdysteroidogenesis are evolutionally conserved between *Bombyx* and *Drosophila*. To assess the function of the candidate genes obtained from our silkworm RNA-seq screening, we conducted *Drosophila* GAL4/UAS-based RNAi screening for fly orthologs of these candidate genes. Via this analysis, we observed that eight genes showed developmental defects when RNAi was driven by *phm*-*GAL4* or *2*-*286*-*GAL4*. Particularly, all RNAi lines of *alas* and *CG33156* showed developmental defects. Real-time PCR and *in situ* hybridization confirmed that *alas* and *CG33156* were predominantly expressed in the RG. Although the other six candidate genes, *Spn6*, *sls*, *CG3106*, *CG34394*, *rab3*, and *CG8420*, showed developmental defects upon RNAi, their expression was either not specific to the PG or very low, thus indicative that they may not be relevant to ecdysone synthesis and/or release.

RNAi animals of *alas* or *CG33156* were still larvae and showed low ecdysteroid titers at 144 h AEL when normal animals have completed metamorphosis. These results indicated that both genes are responsible for proper ecdysteroids synthesis and/or release in the larvae. *alas* encodes δaminolevulinic acid synthase, which catalyzes the formation of aminolevulinic acid from succinyl-CoA and glycine and it is the first step of heme biosynthesis. *CG33156* is a predicted gene with NAD kinase domain and expected to associate with NADP(H) production. Heme and NADP(H) are required for normal cell physiology, and disrupting the production of these molecules caused morphogenetic defects during embryogenesis in vertebrates [25, 26]. However, morphology of the RG in the RNAi larvae seemed to be normal at day 5 and then the RG enlarged and showed enhanced GFP expression at day 8. These results strongly suggested that these genes affected neither morphogenesis nor cell survival of the RG but rather the function of the PG as a steroidogenic organ. Some steroidogenic enzymes such as, Spo, Phm, Dib, and Sad, are members of the cytochrome P450 monooxygenase (CYP) family, which requires heme as a cofactor for electron transfer and NADPH as an electron donor for their enzymatic function. Therefore, *alas* and *CG33156* might be essential factors for CYP function in ecdysteroid synthesis, whereas details remain to be seen. There are two different genes encoding ALAS isozyme in vertebrates [27–29]. One gene (*alas1*) encodes an isozyme ubiquitously expressed and another (*alas2*) encodes an erythrocyte-specific isozyme. In contrast, both the fruitfly and silkworm have a single *alas* gene in respective genomes. In *Drosophila*, expression of *alas* has been reported in seven pairs of oenocytes and two unidentified cells in the anterior during embryogenesis [30]. Furthermore, this study revealed that *alas* was highly expressed in the RG, with low expression was detected in other tissues in wandering larva. Such high expression of *alas* in the PG may help to meet the demand of CYPs during steroidogenesis. In addition, *CG33156* was predominantly expressed in the RG. In humans, there are two types of NADK, cytosolic *NADK1* and mitochondrial *NADK2*, and they are expressed in most human tissues [31, 32]. CG33156 showed high similarity to human NADK1 by BLASTP search (identity = 63.4%, E-value = 5.6e-133). In the Fly Base, there were two more homologs to human NADK (CG6145 to human NADK1, identity = 54.1%, E-value = 1.8e-120; CG8080 to human NADK2, identity = 36.4%, E-value = 2.6e-64). All of them have a conserved NAD kinase domain and may function as a NAD kinase *in vivo*. When *CG33156* were solely knocked down in the RG, the RNAi animals could not make puparium, and neither *CG6145* nor *CG8080* could compensate for the lack of *CG33156* function. Thus, *CG33156* might have a pivotal role in ecdysone synthesis in the RG as a provider of NADP(H). Further precise functional analysis of Alas and CG33156 will provide much insight into the temporal dynamics of ecdysone synthesis and/or release.

In conclusion, our RNA-seq analysis of the *Bombyx* PG and phenotypic analysis of RNAi in *Drosophila* revealed two new players in the regulation of ecdysone synthesis and/or release. The genes analyzed in this study are just the tip of the iceberg, and the foundation we have formed may help to accelerate further elucidation of insect steroid synthesis and release. Particularly, future work purposed to construct the ecdysone transcriptional network in the PG and searching for target genes encoding effector molecules will shed light on the molecular basis of insect hormone production, release, and regulatory mechanisms.

## Supporting information

S1 Table Primer sets for real-time PCR and *in situ* hybridization. (XLSX)

S2 Table Multiple reaction monitoring parameters of Makisterone A. (XLSX)

S3 Table Expression ratios of the top 30 genes in the *B*. *mori* prothoracic gland. (XLSX)

S4 Table List of genes and RNAi lines examined in this study. In the “RNAi line” column, V and N indicate that the RNAi lines were obtained from VDRC and NIG-FLY, respectively.
(XLSX)
